# An economic evaluation of chronic obstructive pulmonary disease clinical pathway in Saskatchewan, Canada: Data-driven techniques to identify cost-effectiveness among patient subgroups

**DOI:** 10.1371/journal.pone.0301334

**Published:** 2024-04-01

**Authors:** John Paul Kuwornu, Fernando Maldonado, Gary Groot, Elizabeth J. Cooper, Erika Penz, Leland Sommer, Amy Reid, Darcy D. Marciniuk

**Affiliations:** 1 Australian Centre for Health Services Innovation and Centre for Healthcare Transformation, Faculty of Health, School of Public Health and Social Work, Queensland University of Technology, Brisbane, Queensland, Australia; 2 Health Quality Council, Saskatoon, Saskatchewan, Canada; 3 Community Health and Epidemiology, University of Saskatchewan, Saskatoon, Saskatchewan, Canada; 4 Kinesiology and Health Studies, University of Regina, Regina, Saskatchewan, Canada; 5 Respirology, Critical Care & Sleep Medicine, The Respiratory Research Centre, University of Saskatchewan, Saskatoon, Saskatchewan, Canada; 6 Stewardship and Clinical Appropriateness, Saskatchewan Health Authority, Regina, Saskatchewan, Canada; 7 Clinical Integration Unit, Saskatchewan Health Authority, Regina, Saskatchewan, Canada; Taipei Medical University, TAIWAN

## Abstract

**Background:**

Saskatchewan has implemented care pathways for several common health conditions. To date, there has not been any cost-effectiveness evaluation of care pathways in the province. The objective of this study was to evaluate the real-world cost-effectiveness of a chronic obstructive pulmonary disease (COPD) care pathway program in Saskatchewan.

**Methods:**

Using patient-level administrative health data, we identified adults (35+ years) with COPD diagnosis recruited into the care pathway program in Regina between April 1, 2018, and March 31, 2019 (*N* = 759). The control group comprised adults (35+ years) with COPD who lived in Saskatoon during the same period (*N* = 759). The control group was matched to the intervention group using propensity scores. Costs were calculated at the patient level. The outcome measure was the number of days patients remained without experiencing COPD exacerbation within 1-year follow-up. Both manual and data-driven policy learning approaches were used to assess heterogeneity in the cost-effectiveness by patient demographic and disease characteristics. Bootstrapping was used to quantify uncertainty in the results.

**Results:**

In the overall sample, the estimates indicate that the COPD care pathway was not cost-effective using the willingness to pay (WTP) threshold values in the range of $1,000 and $5,000/exacerbation day averted. The manual subgroup analyses show the COPD care pathway was dominant among patients with comorbidities and among patients aged 65 years or younger at the WTP threshold of $2000/exacerbation day averted. Although similar profiles as those identified in the manual subgroup analyses were confirmed, the data-driven policy learning approach suggests more nuanced demographic and disease profiles that the care pathway would be most appropriate for.

**Conclusions:**

Both manual subgroup analysis and data-driven policy learning approach showed that the COPD care pathway consistently produced cost savings and better health outcomes among patients with comorbidities or among those relatively younger. The care pathway was not cost-effective in the entire sample.

## Introduction

The global economic and health burdens of chronic health conditions are mounting, and there is an urgent need for cost-effective chronic disease management programs. One example of a chronic disease management program that is rising in popularity is the care pathway (also called the clinical pathway). A care pathway is a structured, multidisciplinary care plan with the following characteristics: a) translates guidelines or evidence into local structures, b) details the steps in a course of treatment or care in a plan, guideline, or protocol, and c) aims to standardize care for a specific clinical problem, procedure or episode of healthcare in a specific population [1]. Saskatchewan, a province in Canada with a population of 1.13 million (2021), has implemented care pathways for several common and resource-intensive health conditions, including prostate cancer, acute stroke, and more recently, chronic obstructive pulmonary disease (COPD). Despite the investments since 2009, there has not been a rigorous evaluation of the cost-effectiveness of care pathways in the province [1, 2].

This study focuses on the economic evaluation of the COPD care pathway in Saskatchewan because an important consideration that speaks to the potential benefits and impact of the intervention is the burden of disease. COPD is the third leading cause of death worldwide, causing 3.23 million deaths in 2019 [3], and ranks as the 6^th^ leading cause of global disability-adjusted life years [4]. COPD accounts for over 50% of chronic respiratory disease prevalence among adults [5], and for an astounding 81.7% of the total number of deaths from chronic respiratory diseases [6].

Previous studies that examined the cost-effectiveness of care pathways among individuals living with COPD used different names for the intervention, including integrated care pathway [7], person-centered care [8], integrated disease management program [9, 10], or respiratory coordinated care program [11]. Most of these studies were based on randomized control trials [8, 9], cluster randomized control trials [12, 13], or observational studies [11, 14, 15]. Different types of economic evaluations were used, including cost-effectiveness analysis [9, 12], budget impact analysis [7], and cost-minimization analysis [11, 16]. The most common effects (outcomes) investigated were quality-adjusted life years (QALYs) [8, 9, 13], 90-day mortality [15], patient’s exercise tolerance [14, 16], and frequency of exacerbations [12, 17]. Most of the studies found care pathways to be cost-effective regarding outcomes such as QALYs [8, 9, 13], hospital readmissions [11, 17], or physical activity [14]. Other studies found care pathways not to be cost-effective for reducing the frequency of exacerbations [12] or 90-day mortality [15].

Similar to our previous synthesis of the pertinent literature [1], two main issues crystalize from the overview of previous studies on the cost-effectiveness of COPD care pathways. First, except for one study [12], all previous studies reviewed presented “conventional” cost-effectiveness analyses. These types of cost‐effectiveness analyses compare the mean difference in health outcomes over the mean difference in costs between alternative treatments. However, decisions based on average cost-effectiveness measures may lead to incorrect treatment recommendations for specific subsets of the population and result in inefficient allocation of resources [18]. Accounting for patient heterogeneity (the variability in costs and/or health outcomes that is related to factors such as sociodemographic characteristics of patients) may help policy-makers target implementation strategies toward subgroups with the most advantageous cost‐effectiveness outcomes [19–22]. Second, while a few studies used observational study design methods, the vast majority of the studies we found were based on clinical trials [14, 15]. Care pathway interventions are generally broad, cutting across multiple care settings and are intended to improve the overall health of the patient [10]. These characteristics make care pathways difficult to be amenable to clinical trials, which are more suitable for tightly controlled interventions [10]. Clinical trial evaluations of care pathways reported in the literature usually tend to focus on only one healthcare sector, such as inpatient hospital care. Additional insights may be gained from observational studies that evaluate real-world implementations of COPD care pathways covering the patient’s experiences across the continuum of care [23].

This study improves upon the existing literature by adopting an observational study design to conduct real-world cost-effectiveness analysis of a COPD care pathway program and assessing the heterogeneities in cost-effectiveness across patient demographic and disease characteristics.

## Methods

### Intervention and study setting

The COPD care pathway began as a collaborative project between various organizations, including the former Regina Qu’Appelle Health Region (RQHR), the Saskatchewan Health Quality Council (HQC), the *LiveWELL* COPD program [24] in the former Saskatoon Health Region, the University of Saskatchewan, Saskatchewan Ministry of Health, and Lung Saskatchewan. The pathway relies on care coordination between local primary care providers, respirologists, respiratory therapists, pharmacists and nurses; with the goals of increasing quality of care and reducing healthcare utilization [1]. The COPD care pathway was implemented in the former RQHR, now called Regina Area of the Saskatchewan Health Authority (SHA), in September 2017. The COPD care pathway consists of a five-step process to provide coordinated patient navigation, including preventions programs, targeted screening, testing, diagnosis, and clinical management of the disease (Table 1) [1].

**Table 1:** Key care elements in the chronic obstructive pulmonary disease (COPD) care pathways program.

Key Care Element	Details
Prevention	Prevention activities such as physical activity, smoking cessation, vaccinations, etc. that are aimed at reducing the risk of COPD^a^ and related complications.
Targeted Screening	Screening to identify individuals at risk using screening posters (both print and electronic) designed for COPD. If high clinical suspicion, individuals are referred for spirometry testing.
Lung Function Testing	Spirometry (mandatory for diagnosis). Testing must meet acceptability and reproducibility criteria per guidelines
Diagnosis	Post bronchodilator FEV1^b^/FVC^c^ < 0.7. In some instances, FEV1/FVC < lower limit of normal may be used. If additional investigations are required, referral to a specialist is considered.
Clinical Management	Medications, Patient self-management (the patient being able to understand and recognize COPD exacerbations and have a COPD Action Plan), Chronic disease rehab, and Oxygen therapy.

^a^ COPD: chronic obstructive pulmonary disease

^b^ FEV1: forced expiratory volume in 1 second

^c^ FVC: forced vital capacity

After a diagnosis of COPD is confirmed, clinical management of the disease includes both pharmacologic and non-pharmacologic therapies, including oxygen therapy, self-management with COPD Action Plan (developed by a patient and their healthcare provider), medication management, vaccination, and pulmonary rehabilitation. The pulmonary rehabilitation program offers supervised aerobic exercise training and COPD education aimed at relieving symptoms, slowing disease progression, improving quality of life, and decreasing hospital and unscheduled doctor visits, outcomes previously documented to improve with participation in pulmonary rehabilitation [25]. Patients were referred into the COPD care pathway from multiple sources, including family physicians, respirologists, respiratory therapists, internal medicine physicians, acute care nurse navigators, and intermediate care paramedics. Further details on the development and implementation of the COPD care pathway was previously published [1]. Only patients who provided written consent for their records to be linked with other administrative health data were included in the study.

Saskatchewan provides universal health care coverage for acute care visits (emergency department [ED] and hospital) and physician visits for the majority (about 99%) of the population except for persons covered by the federal government (i.e., members of the Canadian Armed Forces and Royal Canadian Mounted Police [RCMP], registered Indigenous status [First Nations and Inuit], people in federal correctional facilities, as well as individuals with refugee/asylum status). The province also provides supplemental prescription drug coverage for eligible individuals over age 65 years and for those with limited incomes.

### Data sources

The study used population-based administrative health data from Saskatchewan, including emergency department (ED) records, hospital discharge abstracts, physician billing claims, outpatient prescription drug dispensation records, and population registration files. These data sets cover all residents of Saskatchewan regardless of whether the province or the federal government pays for the care. The data sets were deterministically linked together using an anonymized personal health identification number to create a longitudinal healthcare utilization record across the continuum of care for everyone included in the study. Access to the data sets was granted on June 14, 2021. Analyses were conducted at the secure data laboratory of the HQC, with the analyst having no access to personal identifying information of study participants. Ethics approval for the research was received from the Saskatchewan Health Authority Research Ethics Board (REB-20-69).

### Intervention and control groups

The intervention group comprised of individuals aged 35+ years who were diagnosed with COPD and recruited into the COPD care pathway program in Regina (population 226,404) between April 1, 2018 and March 31, 2019. The control group included individuals 35+ years who were diagnosed with COPD and lived in Saskatoon (population 266,141) between April 1, 2018 and March 31, 2019. Both the intervention and the control groups met the criteria of a validated case definition for COPD [26]: (1) a hospital discharge abstract and/or physician visit with a diagnosis of COPD. Individuals with COPD diagnosis were identified using the International Classification of Diseases, 10^th^ Revision, Canada (ICD-10-CA) codes J41, J42, J43 or J44 in the hospital discharge abstracts; whilst ICD-9 codes 491, 492 or 496 were used to identify individuals in the physician billing claims [27]. This case definitions has been used in previous studies [28, 29]. COPD patients residing in Saskatoon were selected as controls because Saskatoon and Regina are the two main urban centers in Saskatchewan (population > 200,000 in each center), and together account for about half of the provincial population.

The index date for patients in the COPD care pathway program was the date of recruitment. Referrals were made during contact with care providers for COPD-related reasons. For patients in the control group, the index date was the earliest hospitalization or physician visit date for COPD-related reasons in the corresponding recruitment period.

### Costs

#### Healthcare costs

Healthcare costs were calculated for inpatient hospitalizations, ED visits, physician visits (both general practitioners and specialists), and outpatient prescription drug dispensations in the one-year period before and after the index date. Hospital costs were estimated using the provincial average direct costs per weighted case. This standard methodology developed by the Canadian Institute for Health Information for all Canadian provinces, uses the product of resource intensity weights and provincial cost of a standard hospital stay to estimate cost of inpatient hospital stays at the patient level [30]. For the ED cost component, average costs per ED visit were obtained from the Ministry of Health and applied to ED visits during the follow-up period. The cost of a physician visit was the amount billed by the physician to the Saskatchewan Ministry of Health, the sole source of physician fee-for-service reimbursement in the provincial healthcare insurance plan. Prescription drug costs were based on the price of the active substance plus a dispensing fee and any applicable markups.

Costs were adjusted for inflation using the health and personal care (hospitalization, physician visits, ED visits) and the Medicinal and pharmaceutical products (prescription drugs dispensations) items of the Saskatchewan consumer price index [31] and expressed in 2019/20 constant Canadian dollars. The methods we used to estimate healthcare costs were applied in previous studies using Saskatchewan administrative health data [28, 29]. All healthcare costs were calculated from the perspective of a single public payer (i.e., the Saskatchewan Ministry of Health), excluding individual out-of-pocket expenditures such as copayments.

#### Cost of implementation

Direct costs related to the development, implementation, and maintenance of the intervention program were tracked and allocated to patients in the COPD care pathway. These costs included patient recruitment, personnel, equipment, and facility rentals (see S1 Table for details). These costs were added to the total healthcare costs of patients who were in the care pathway.

### Effects

Similar to costs, we measured effectiveness both in the one-year period before and after the index date. The measure of effectiveness was the number of days patients stayed within one-year period without experiencing acute COPD exacerbation. COPD exacerbations negatively affect patients’ quality of life and increase the risk of death [32]. We calculated the number of days patients experienced acute COPD exacerbations and subtracted these from 365 days. ICD-10-CA codes J41, J42, J43, J44 were used to identify episodes of acute COPD exacerbations in inpatient hospital and ED records, whilst ICD-9 codes 491, 492, 496 were used to identify episodes of acute COPD exacerbations in physician visit records. These codes were validated and previously used to identify episodes of COPD exacerbations from administrative health data in Saskatchewan [29]. The ICD-10-CA codes must be in the most responsible diagnosis field, or a diagnosis of an acute lower respiratory tract infection in the most responsible diagnosis field and a diagnosis of other COPD (ICD-10-CA code J44) in the second diagnosis field. The ICD-9 codes must be accompanied by outpatient dispensation of drugs used to treat acute exacerbations of COPD, including antibiotics, systemic corticosteroids, short-acting beta agonists (SABAs), and SABAs combined with anticholinergics within 2 days of the physician visit. Similarly, the number of days patients stayed in the one-year period prior to the index date without experiencing acute COPD exacerbation was calculated.

### Patient and disease characteristics

The patient demographic characteristics included in the analysis were sex (i.e., male or female) and age group (i.e., 35–45, 46–55, 56–65, 66–75, 76+). Patients’ disease characteristics were estimated using a list of the 20 most common health conditions included in the Charlson comorbidity index [33] for our cohort; including health conditions such as congestive heart failure, depression, etc. These comorbid conditions were defined using ICD-9 and ICD-10-CA codes and were based on diagnoses in the hospital discharge abstract and the physician billing claims data. All variables were defined as of the index date except for the comorbid conditions, which were defined using data from the three-year period prior to the index date.

### Statistical analysis

#### Propensity score matching

We used propensity score matching to control for confounding and minimize the risk of bias in our analyses. All the listed demographic characteristics and 20 health conditions were included in the propensity score models. Individuals in the COPD care pathway group were matched to individuals in the control group using propensity score matching. We used one-to-one matching on the propensity scores with the nearest neighbor matching algorithm without replacement to form matched pairs of treated and untreated individuals. Standardized mean difference (SMD) was used to ascertain balance between groups, with SMD values of 0.1 and lower indicating balance.

#### Cost-effectiveness analysis

Various analyses were undertaken to ascertain the effectiveness and cost-effectiveness of the COPD care pathway. Adjusted mean differences were calculated to show the average effect of intervention (i.e., effectiveness) on the outcome and healthcare cost, while the incremental cost-effectiveness ratio (ICER) and average net monetary benefit were used to ascertain cost-effectiveness. Costs and effects were not discounted due to the one-year follow-up used in the study. The cost-effectiveness analyses relied on a causal forest methodology [34] called cost-effectiveness analysis (CEA) forests developed by Bonander and Svensson [35]. The method was used to calculate the average cost-effectiveness in the entire sample and cost‐effectiveness across patient subgroups. While cost-effective subgroups can be identified manually, it may also be advantageous to use data-driven techniques to search for treatment allocation rules. These methods of learning treatment assignment policies, sometimes referred to as policy learning [36], are relatively new yet highly impactful area of research. The CEA forests approach is advantageous because it can (1) learn complex patterns of heterogeneity from the data without explicit assumptions on functional form and (2) identify subgroups automatically. We used both manually defined and data-driven (i.e., policy learning) approaches to identify subgroups where the COPD care pathway is more likely to be cost-effective. We also estimated the expected welfare gain [35] per population member when treatment allocation policy suggested by the data-driven approach were implemented as opposed to giving everyone the (1) control treatment or (2) the COPD care pathway.

Healthcare decision makers might be guided in estimating their willingness-to-pay (WTP) by considering the potential cost savings of the intervention. Previous Canadian studies have estimated an average cost of $955 per day of hospitalization for severe exacerbations [37] or an average of $3,036 for an acute episode of COPD exacerbation [38]. We considered these resource use estimates to select WTP threshold values ranging between $1,000 and $5,000/exacerbation day averted in our study.

#### Control of confounding and manual subgroup analysis

The CEA forest model included the following as covariates: age, sex, the Charlson comorbidity score, and the 20 most common health conditions in the study cohort. We included these variables in the models because some of them potentially had residual confounding after matching since the SMD were not zeroes. Also, healthcare costs and the effects (i.e., number of days patients stayed without experiencing acute COPD exacerbation) in the one-year period prior to the index date were included in the CEA forest model as covariates. These latter variables were important because we were unable to measure severity of COPD cases. Controlling for healthcare costs and effects in the one-year period prior to the index date would likely minimize any potential confounding effects that may arise from differences in disease severity between the study groups.

The manually defined subgroup analyses were based on Charlson comorbidity index (score > = 2 vs score < 2), age (age > 65 years vs Age < = 65 years), and sex (male vs female). The cut-point for the comorbidity index allows for comparison between patients who had additional health conditions and those who only had COPD. Statistics Canada usually defines elderly persons as anyone over 65 years of age. Our age cut point allows for comparison between the elderly and younger patients.

#### Uncertainty and sensitivity analysis

To quantify uncertainty in the estimates, bootstrapping was applied to the doubly robust scores with replacement after training the forest and was used to construct confidence intervals for ICERs as well as the cost‐effectiveness planes [35]. The cost-effectiveness acceptability curve (CEAC) was also used to quantify uncertainty in the results. CEAC illustrates the probability that the intervention group (i.e., COPD care pathway) is cost-effective compared with the control group.

All analyses were performed using R (version 4.1.0, R Foundation for Statistical Computing, Vienna, Austria). Statistical significance was determined by a p-value of <0.05.

## Results

**Fig 1:**
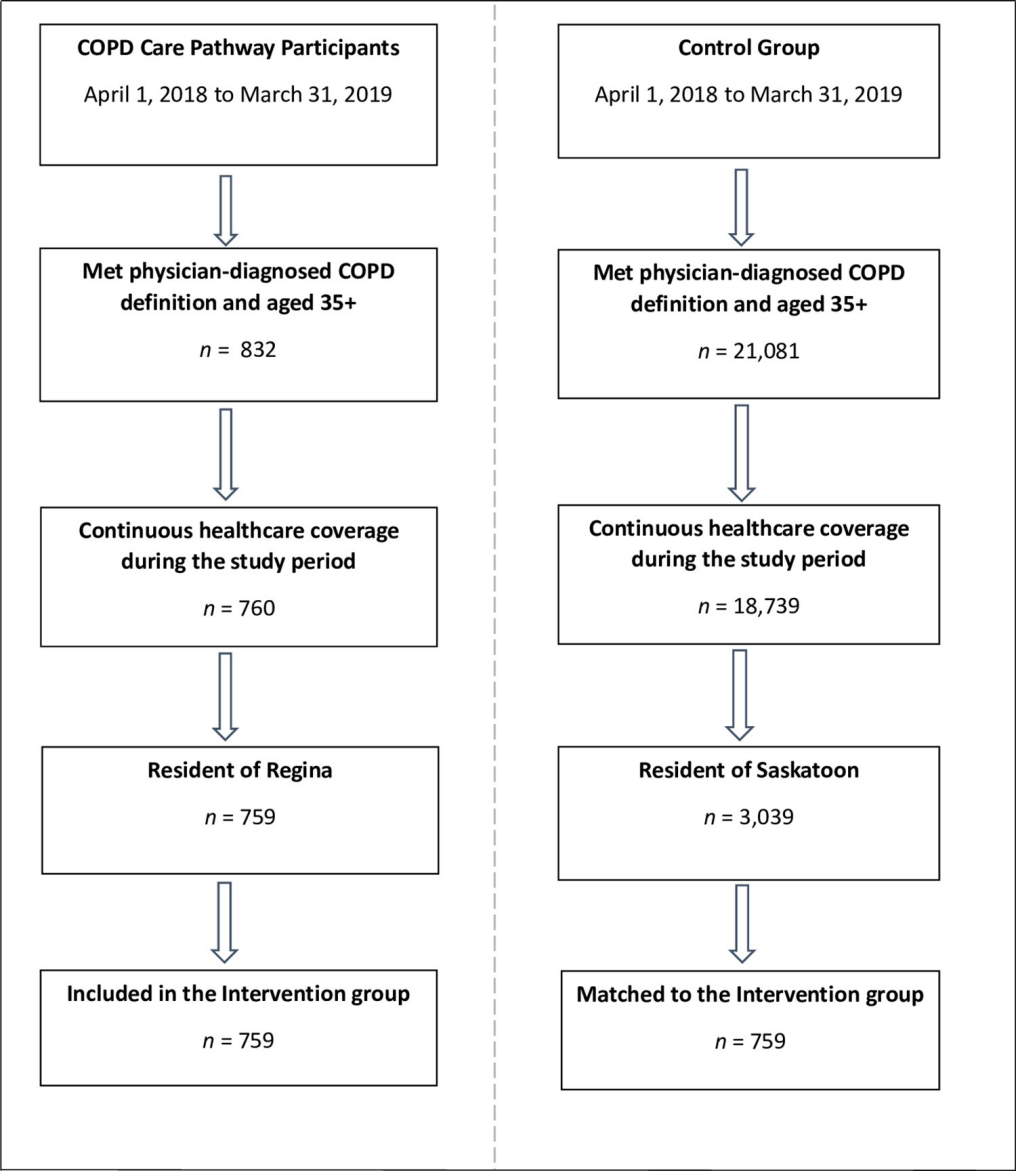
Study cohort flow diagram. COPD: chronic obstructive pulmonary disease. FY: fiscal year.

A total of 759 individuals with COPD who had continuous health coverage and met the age requirements between April 1, 2018 and March 31, 2019 were included in the COPD care pathway group. After the exclusion criteria were applied (i.e., not meeting the COPD case definition, not having continuous provincial health insurance coverage, etc.,) the remaining individuals were eligible to be included in the study as controls (Fig 1).

Similar to our previous study [1], after one-to-one matching using propensity scores, all individuals in the COPD care pathway group (N = 759) were matched to the control group (N = 759). Overall, 1,518 individuals were included in the analysis (mean [SD] age, 62.6 [13.6] years; 857 females [56.4%]). All covariates except hypertension uncomplicated were balanced between the study groups based on the SMDs after matching (Table 2).

**Table 2:** Comparison of baseline characteristics between treatment and control subjects in the unmatched and matched samples.

	Before Matching			After Matching		
Variable	COPD^a^ pathway (*N* = 759)	Control (*N* = 3,038)	SMD^b^	COPD pathway (*N* = 759)	Control (*N* = 759)	SMD^c^
%						
**Age**			0.36			0.05
35–45	12.1	4.6		12.1	12.6	
46–55	14.4	11.9		14.4	15.5	
56–65	30.6	26.7		30.6	29.5	
66–75	23.7	27.9		23.7	24.4	
76+	19.2	28.9		19.2	17.9	
**Female**	56.0	49.7	0.13	56.0	56.9	0.02
**Comorbid Health Conditions**						
Cardiac Arrythmia	3.3	2.1	0.08	3.3	4.2	0.05
Congestive Heart Failure	7.4	7.7	0.01	7.4	9.2	0.07
Coagulopathy	2.4	2.2	0.01	2.4	3.2	0.05
Deficiency Anemia	2.6	0.7	0.15	2.6	2.1	0.04
Depression	10.7	9.3	0.04	10.7	9.9	0.03
Diabetes Complicated	3.4	4.1	0.04	3.4	4.5	0.05
Diabetes Uncomplicated	6.5	3.3	0.15	6.5	7.2	0.03
Drug Abuse	2.1	2.8	0.04	2.1	1.6	0.04
Fluid and Electrolyte Disorder	3.7	2.7	0.05	3.7	4.2	0.03
Hypertension Uncomplicated	37.0	32.0	0.11	37.0	43.1	0.12
Hypothyroidism	5.0	7.5	0.10	5.0	3.8	0.06
Liver Disease	1.2	0.9	0.03	1.2	1.2	<0.01
Other Neurological Disorder	1.6	1.3	0.03	1.6	2.0	0.03
Pulmonary Circulation Disorder	1.8	5.2	0.18	1.8	2.0	0.01
Peripheral Vascular Disorder	1.6	1.4	0.01	1.6	2.1	0.04
Psychosis	2.0	5.1	0.17	2.0	1.6	0.03
Renal Failure	4.3	3.1	0.07	4.3	6.3	0.09
Rheumatoid Arthritis/ Collagen Vascular Disease	2.2	4.5	0.12	2.2	2.1	0.01
Solid Tumor without Metastasis	4.2	5.3	0.05	4.2	3.6	0.03
Valvular Disease	2.4	2.6	0.02	2.4	3.3	0.06

Values reported in the table are either percentages (%) or SMD

^a^ COPD: chronic obstructive pulmonary disease

^b^ SMD: standardized mean difference, was used to ascertain balance between groups, with SMD values of 0.1 or lower indicating balance.

^c^ One-to-one matching on the propensity scores with the nearest neighbor matching algorithm without replacement was used to form matched pairs of treated and untreated individuals.

The cost-effectiveness results for the entire sample and sub-groups are reported in Table 3. The estimates indicate that the COPD care pathway was not cost-effective on average in the entire sample; using WTP threshold values ranging between $1,000 and $5,000/exacerbation day averted. The COPD care pathway was dominant among patients with comorbidities whilst the control treatment dominates the care pathway among patients without comorbidities, at the WTP threshold of $2,000/exacerbation day averted. The COPD care pathway was dominant among patients younger than 66 years whilst the control treatment dominates the care pathway among patients aged 66 years and above, at the WTP threshold of $2,000/exacerbation day averted. The control treatment dominates the COPD care pathway in both male and female subgroups, at the WTP threshold of $2,000/exacerbation day averted.

**Table 3:** Results of the cost-effectiveness analyses for the entire sample and for the manually defined subgroups of Charlson comorbidity index, age, and sex.

	Estimate (95% CI)	Pr (cost-effective)
Full sample		
Average effect on the outcome	0.04 (-1.73–1.49)	NA
Average effect on cost	217 (-2534.47–2157.03)	NA
Incremental cost-effectiveness ratio	5268.64 (2047.81–8936.46)	NA
Average net monetary benefit (WTP: $2,000)	-134.64 (-4440.76–4439.62)	0.48
Average net monetary benefit (WTP: $1,000)	-175.83 (-308.94–3156.24)	0.46
Average net monetary benefit (WTP: $5,000)	-11.07 (-9630.35–7479.02)	0.50
Subgroup: Charlson Index > = 2		
Average effect ^a^ on the outcome	1.19 (-0.95–3.19)	NA
Average effect on cost	-163.60 (-6738.25–3802.73)	NA
Incremental cost-effectiveness ratio	Dominant ^b^	
Average net monetary benefit (WTP: $2,000)	2535.01 (-3785.42–10874.59)	0.75
Subgroup: Charlson Index < 2		
Average effect on the outcome	-0.90 (-3.87–0.83)	NA
Average effect on cost	530.02 (-818.54–1934.88)	NA
Incremental cost-effectiveness ratio	Dominated ^c^	
Average net monetary benefit (WTP: $2,000)	-2329.97 (-8850.65–1830.89)	0.20
Subgroup: Age > = 66 years		
Average effect on the outcome	-1.84 (-5.93–0.82)	NA
Average effect on cost	3053.47 (106.92–6042.04)	NA
Incremental cost-effectiveness ratio	Dominated	
Average net monetary benefit (WTP: $2,000)	-6738.39 (-15836 –- 109.92)	0.04
Subgroup: Age < 66 years		
Average effect on the outcome	1.44 (0.41–2.81)	NA
Average effect on cost	-1889.97 (-6350.87–556.07)	NA
Incremental cost-effectiveness ratio	Dominant	
Average net monetary benefit (WTP: $2,000)	4770.79 (1003.33–10274.56)	0.99
Subgroup: Male		
Average effect on the outcome	0.39 (-1.70–2.06)	NA
Average effect on cost	1060.47 (-1402.12–3753.31)	NA
Incremental cost-effectiveness ratio	2690.87 (11.41–414482.42)	NA
Average net monetary benefit (WTP: $2,000)	-272.27 (-5234.67–4525.98)	0.48
Subgroup: Female		
Average effect on the outcome	-0.23 (-3.13–1.66)	NA
Average effect on cost	-433.53 (-5468.55–2170.05)	NA
Incremental cost-effectiveness ratio	1876.69 (-733.11–1657947.50)	NA
Average net monetary benefit (WTP: $2,000)	-28.48 (-6648.55–6604.35)	0.48

CI: confidence interval

Pr: probability

WTP: willingness to pay

^a^ Average effects on the outcome and cost were calculated using adjusted mean differences between groups

^b^ Dominant is when COPD care pathway demonstrated cost savings and improved outcomes compared to control

^c^ Dominated is when COPD care pathway demonstrated higher costs and worst outcomes compared to control

**Fig 2:**
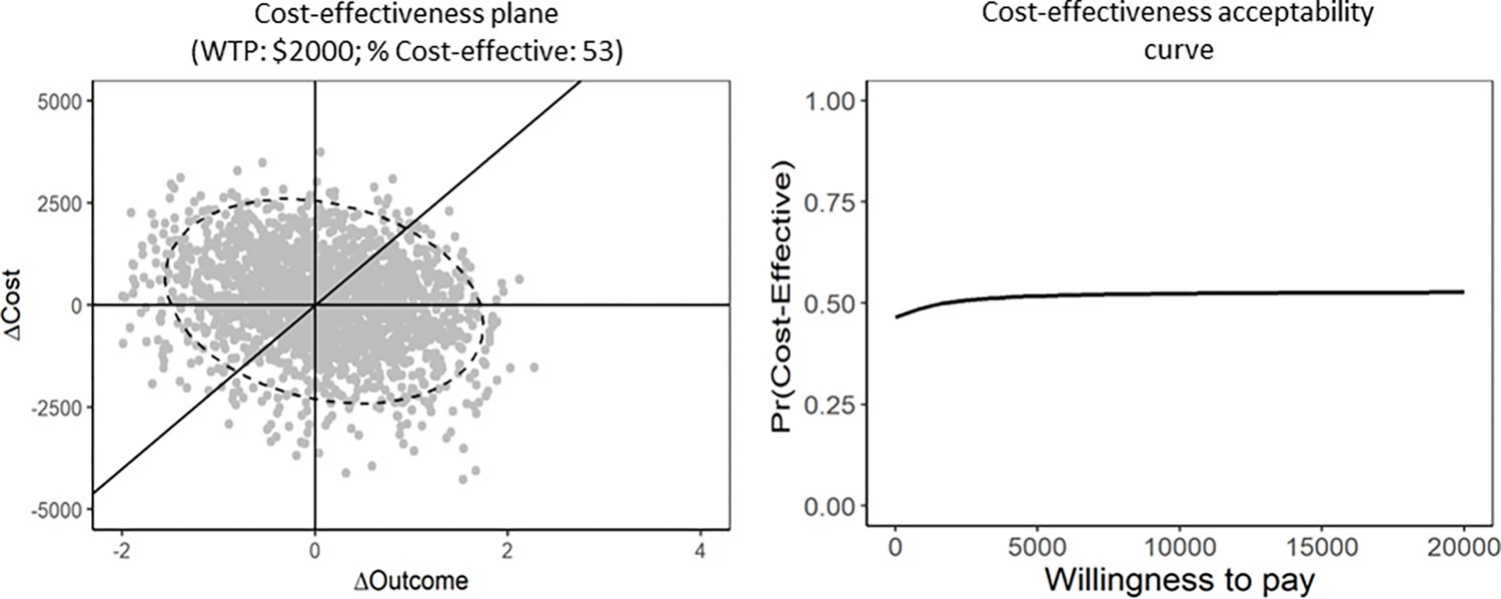
Cost-effectiveness plane and cost-effectiveness acceptability curve for the entire sample. COPD: chronic obstructive pulmonary disease. WTP: willingness to pay.

The uncertainty around ICERs reported for the entire sample was plotted on the cost-effectiveness plane using bootstrapping of the doubly robust scores with replacement (Fig 2). As shown in the cost-effectiveness plane, the bootstrapped replicates of ICERs were almost equally located in all four quadrants, with only 55.5% probability that the care pathway was cost-effective at WTP threshold of $2,000/exacerbation day averted (Fig 2). The Cost-effectiveness acceptability curve indicates the probability that the COPD care pathway is cost-effective compared with controls, for a range of WTP values that a decision maker might consider as the maximum cost they are willing to pay to avert one hospital day of acute COPD exacerbation. For example, given a maximum WTP of $10,000/exacerbation day averted, the probability that the COPD care pathway is cost-effective compared with control treatment is 0.58 (Fig 2).

**Fig 3:**
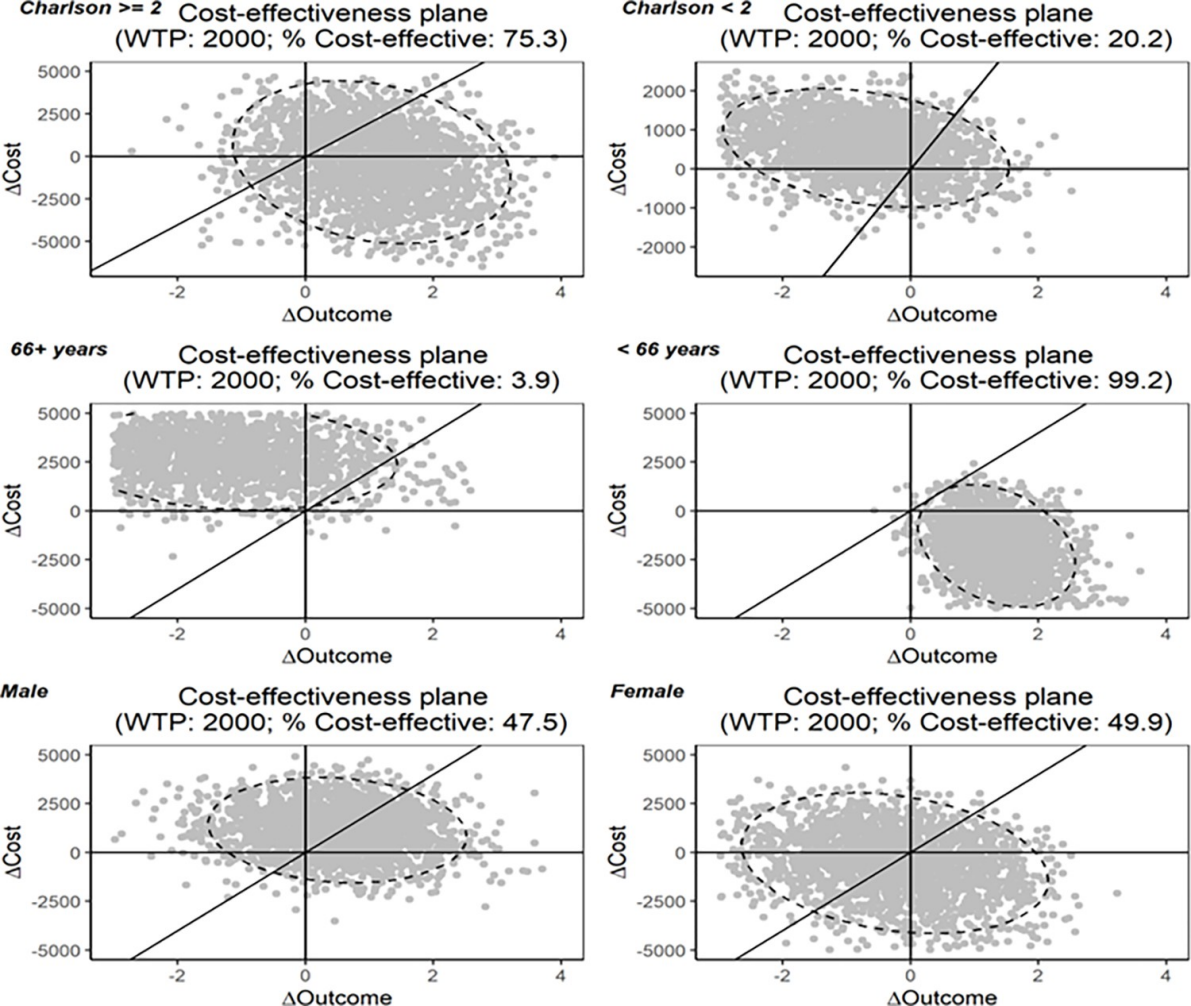
Cost-effectiveness planes, by manually defined subgroups of Charlson comorbidity index, age, and sex. COPD: chronic obstructive pulmonary disease. WTP: willingness to pay.

Similarly, the uncertainties in ICERs by subgroups are reported in the cost-effectiveness planes in Fig 3. Overall, the uncertainties in ICERs varied by patient subgroup.

**Fig 4:**
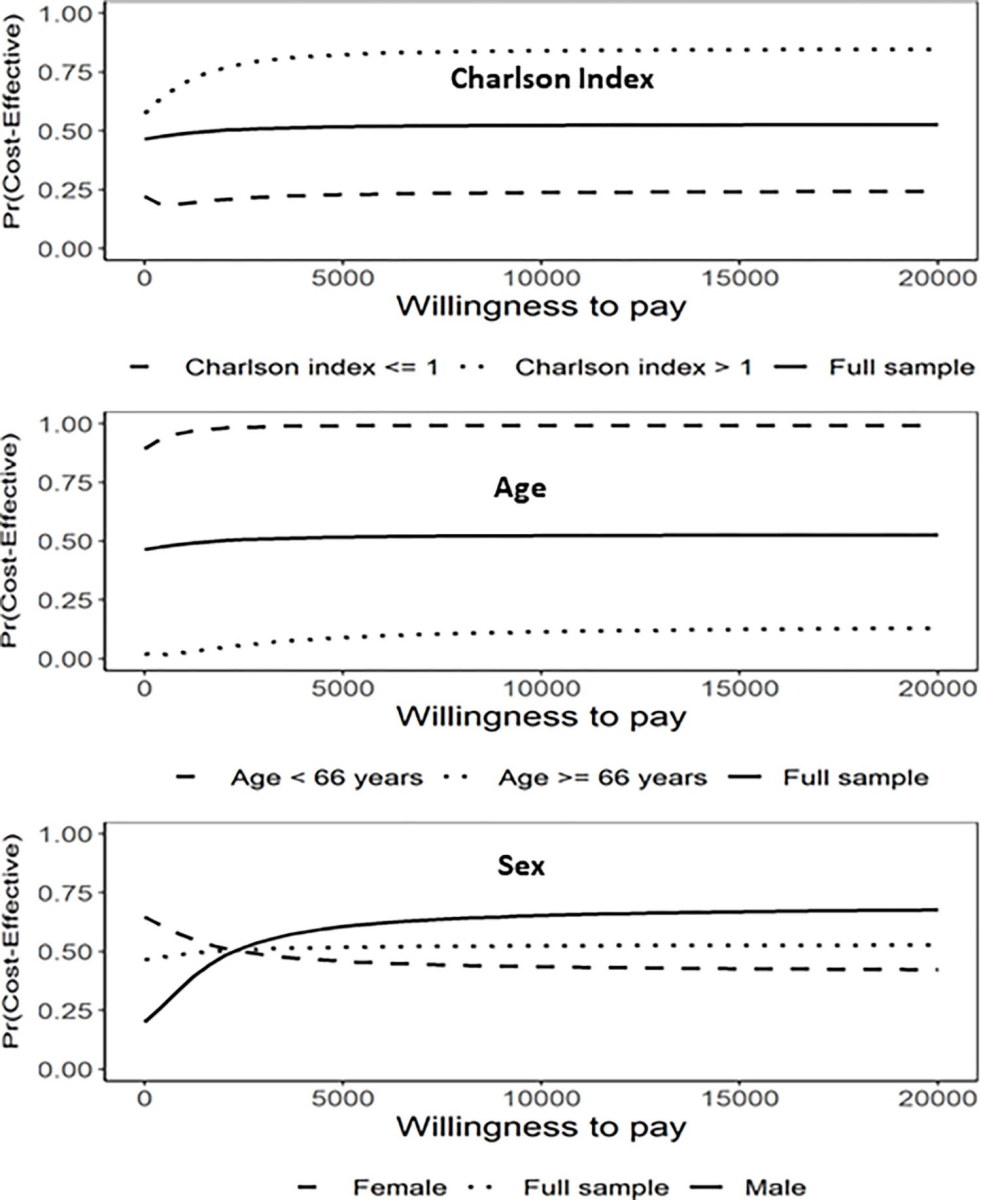
Cost-effectiveness acceptability curves, by manually defined subgroups of (a) Charlson comorbidity index, (b) age, and (c) sex. COPD: chronic obstructive pulmonary disease.

CEACs were used to quantify the uncertainties in cost-effectiveness by subgroups and reported in in Fig 4. Overall, the uncertainties in cost-effectiveness differed by patient characteristics.

**Fig 5:**
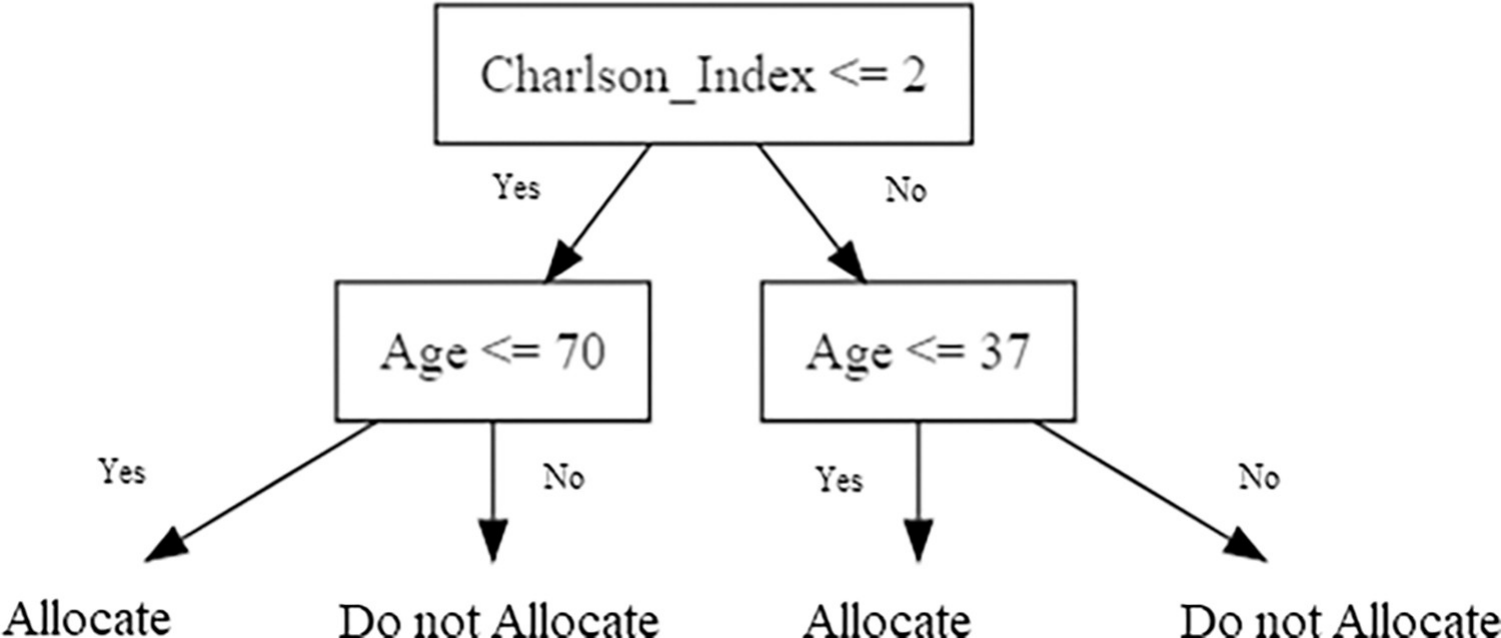
Targeted treatment policies suggested by automated policy learning. COPD: chronic obstructive pulmonary disease.

One of the main rationales of empirical analysis of observational cost-effectiveness studies is to inform policy decisions on personalized treatment allocations based on observable covariates. Fig 5 shows the treatment allocations suggested by the data-driven policy learning approach we implemented, with the accompanying expected welfare gains. The treatment allocation suggested by the algorithm is to allocate the COPD care pathway to patients younger than 71 years with not more than two comorbid conditions or to patients younger than 38 years with more than two comorbid conditions (Fig 5). Compared to recommending the care pathway for all COPD patients, this personalized treatment allocation will allocate 63% of the patients to the care pathway and would result in an estimated welfare gain per population member of $4,901 ($1,807 –$7,996) (Table 4).

**Table 4:** Estimates of the expected welfare gain from implementing targeted treatment policies.

	Expected Welfare Gain (95% CI)
Average NMB: New-for-all vs Control-for-all	$17 (-$4225 –$4258)
Average NMB: Suggested policy vs Control-for-all	$4918 ($2037 –$7799)
Difference in NMB: Suggested vs. New-for-all	$4901 ($1807 –$7996)
Proportion who gets new treatment suggested by policy	0.63

CI: confidence interval

NMB: net monetary benefit

The results are based on the willingness to pay (WTP) threshold values of $2000. Values are expressed in 2020 Canadian dollars.

## Discussion

### Main findings

This study provided a comprehensive cost-effectiveness analysis comparing enrollees in a COPD care pathway to an independent control group. In the context of the overall sample, the COPD care pathway was not cost-effective compared to the control treatment, using WTP threshold values ranging between $1,000 and $5,000/exacerbation day averted. The results from the manual subgroup analyses revealed that the COPD care pathway was dominant (i.e., demonstrated cost saving and improved outcomes) among patients with comorbidities and individuals aged 65 years or younger. The data-driven approach recommended the pathway to patients with fairly similar demographic and disease profiles as those identified in the manual subgroup analysis.

The clinical management component of the COPD care pathway contains several interventions, including oral and inhaled medication management, a COPD Action Plan, chronic disease exercise rehabilitation, and oxygen therapy as required. It is probable some of these interventions may have heterogenous effects among individual subgroups. This may in part explain why the COPD care pathway was dominant for subgroups such as those with comorbidities. Older patients usually have more comorbidities compared to younger patients. Thus, since the care pathway was dominant for those with comorbidities, one might expect it to also be dominant for the elderly. However, the COPD pathway was dominant among individuals aged 65 years or younger, not older patients above age 65 years. This finding may be explained by the fact that the relationship between comorbidities and age is not linear in our study sample. In fact, a number of the younger patients (35–65 years) also had comorbidities. Secondly, it is possible younger individuals may be more adherent to the prescribed exercises in the chronic disease exercise rehabilitation component of the care pathway.

Acknowledging the reality that COPD is a heterogenous disease with various severities and patient phenotypes, it is perhaps not unexpected that benefit from the COPD care pathway was similarly heterogenous. These differences deserve further exploration in order to appropriately align benefit from the COPD care pathway with the patient population most likely to benefit. Further understanding these findings would also facilitate optimal use of limited resources and expertise.

### Comparison with other studies

High-quality studies that evaluate cost differences and variability in cost-effectiveness in COPD interventions by patient characteristics are lacking, particularly those unique to the Canadian context [39]. These types of studies provide important information on the economic burden of the disease and provide understanding on the variations in resource use by various patient groups; all of which are critical for healthcare planning and health resource allocation. Previous studies that examined the cost-effectiveness of COPD care pathways mostly focused on outcomes such as quality-adjusted life years [8, 9, 13] or exercise tolerance [14, 16]. Majority of the studies found COPD care pathways to be cost-effective in relation to QALYs outcome [8, 9, 13]. The only study we found that examined the cost-effectiveness of care pathways in relation to COPD exacerbations was a Dutch randomized controlled trial [12]. Similar to our conclusion regarding the entire sample, the study [12] did not find the intervention to be cost-effective.

### Strengths and weaknesses

This study used advanced statistical methods to conduct real-world cost-effectiveness analysis of a COPD care pathway program and assess the heterogeneities in cost-effectiveness across patient demographic and disease characteristics. Particularly, the use of two complementary approaches (i.e., manual subgroup analysis and data-driven policy learning) provided a nuanced assessment of the heterogeneous effects of the COPD care pathway.

Despite these strengths, the results of the study should be interpreted considering the following limitations. First, a common limitation of studies that use population-based administrative health data is the inability to include all potential confounders such as smoking status, physical activity, body mass index, and spirometry testing results (i.e., FEV_1_/FVC). The latter is used to assess the severity of airflow obstruction in COPD. Unfortunately, these variables were not routinely collected in the data sources we used, thus could not be included in our study. Notwithstanding, we controlled for a comprehensive list of comorbid conditions that may have differential impacts on health outcomes and healthcare costs.

Second, the study used a one-year follow-up period due to the recency of the COPD care pathway implementation and the lag between processing and release of the administrative health data sets. Because of this practical reality it is likely that some of the benefits of the intervention will accrue over a longer period. With the recent and phased implementation of the intervention (beginning in September 2017) it is possible benefits may further increase and become magnified with time.

Third, it is vital to fully appreciate that there are other endpoints and outcomes associated with optimal management of COPD. While frequency and duration of acute COPD exacerbations are important, other patient-centered symptoms such as shortness of breath and activity limitation are equally important to individual patients. The latter endpoints of shortness of breath and activity limitation were not included in our study because they were not routinely collected in the administrative health databases.

Finally, cost-benefit analysis (CBA) could be an appropriate economic evaluation method for our study, particularly if there were opportunities to broaden the scope of assessment of the costs and benefits of the intervention. CBA answers questions about value for money and provides guidance on deciding priorities for funding at the societal level. Consequently, CBA usually adopts broader societal perspective in assessing the costs and benefits of an intervention. We used CEA as our evaluation method since our study was conceived from the healthcare payer/decision-maker’s perspective, and we were limited to using health outcomes and costs contained in provincial health administrative data in Saskatchewan. For example, we did not have information to include items such as out-of-pocket costs and loss of productivity in our cost estimates. Again, we adopted the CEA over CBA because of challenges of monetizing the outcome of our study. Although we found Canadian studies that estimated average cost per day of hospitalization for severe exacerbations [38] and average cost per episode of COPD exacerbation [39], these do not match our outcome of interest. Our outcome measure was COPD exacerbation days averted, without restriction to severe exacerbations. Instead, we considered these resource use estimates reported in the studies as a guide for selecting the range of the decision maker’s WTP threshold. Similar to a previous study [40], we reasoned that healthcare decision makers might be guided in estimating their WTP by considering the potential cost savings of the intervention, which may be reflected in these cost estimates reported in the two studies cited above. Studies that use the net-benefit framework for CEA adopt different approaches for using WTP thresholds to monetize health benefits. Like our study, some studies selected their WTP thresholds based on resource use [40], whilst others use hypothetical ranges of WTP thresholds [41] or ranges of WTP threshold values close to the ICER [42]. The impacts of these choices on cost-effectiveness conclusions can be examined in future studies. Also, future studies can explore the use of CBA in evaluating interventions such as the COPD care pathway. This will enable value for money comparisons between the intervention and other interventions, even beyond the health sector, in order to facilitate priority setting at the societal level.

## Conclusions

Overall, the COPD care pathway was not cost-effective in comparison to the control treatment in the entire sample. However, both manual subgroup analysis and data-driven policy learning approaches showed that the COPD care pathway consistently produced cost savings and better health outcomes among patients with comorbidities or among those relatively younger. This suggests that instead of recommending the care pathway to all COPD patients, personalized treatment allocation may provide greater value. However, the feasibility and practical implications of selectively recommending the care pathway to patient subgroups may require further considerations, which were beyond the scope of this study.

## Supporting information

S1 Table COPD care pathway program cost. (DOCX)
