# Supplementary material for: An economic evaluation of chronic obstructive pulmonary disease clinical pathway in Saskatchewan, Canada: Data-driven techniques to identify cost-effectiveness among patient subgroups
Source: PLoS One. 2024 Apr 1;19(4):e0301334. doi: 10.1371/journal.pone.0301334 (PMC10984414; doi:10.1371/journal.pone.0301334)
Supplement: S1 Table — (DOCX) [file pone.0301334.s001.docx]

Table S1. COPD care pathway program cost

| **Resource/Item** | **Cost** | **Notes on cost calculation** |
| --- | --- | --- |
| **Patient Recruitment** |  |  |
| Diagnostic testing - Spirometry | $41,318.55 | Cost of full ($40.95) spirometry test per patient^a^ |
| Recruitment materials (posters, VBI cards, etc.) | $399.09 | VBI cards - $195.50 + COPD posters - $203.59 |
| Spirometry Interpretation Training | $16,523.91 | Funded by a mix of public and private sector partners |
| **Personnel^b^** |  |  |
| Respiratory Educator | $513,534.11 | 4 FTEs @ $39.674 per hour and 1 FTE @ $42.848 per hour. |
| Social workers | $634.34 | 14* 1 hour lecture @ $45.31 |
| Clerical/administrative | $226,262.40 | CUPE Office Administrative Assistant @ $22.20 per hour. |
| CDPM manager | $114,653.88 | OOS pay band 5 step 6. |
| Exercise therapist | $162,023.50 | 0.5 FTE @ $44.595 per hour and 1 FTE @ $41.291 per hour. |
| Dietitian | $132,975.02 | 1 FTE @ $52.188 per hour. |
| LPNs | $92,976.52 | 1 FTE @ $36.49 per hour. |
| Pharmacist | $871.21 | 14 * 1 hour lecture @ $62.229 |
| FIM leader | $585.94 | 14 * 1 hour lecture training all staff over two days sessions |
| Smoking Cessation Counsellor | $871.21 | 14 * 1 hour lecture @ $62.229 |
| Mental Health Counsellors | $1,214.36 | 28 * 1 hour lecture @ $43.37 |
| Patients & Family Advisors (PFAs) | $437.54 | 50 hours between 12 PFAs |
| SHC RN | $35,712.77 |  |
| SHC Paramedic | $23,668.12 |  |
| Certified Respiratory Educator Course | $24,625.00 | 17*$625 (COPD) + 6*$1875 (CRE) +5*$550 (Spirotrec) |
| **Equipment & Materials** |  |  |
| Spiro kits | $9,147.88 | 10 new equipment @ $73,183.00 (flow sensors, calibration syringes,  software and online support, network software,  offline mode software) depreciated over 8 years^c^ |
| Exercise bands | $11,300.70 |  |
| Projectors | $5,306.00 |  |
| Sat monitors | $62,000.00 | 1000 sat monitors given to patients who have  oxygen concerns |
| Portable concentrators | $6,963.47 | 8 portable concentrators with power cartridge  and desktop chargers @ $34,817.36, depreciate over 5 years^d^ |
| High flow pressure regulators | $1,080.00 | 12 high flow pressure regulators @ $90 each |
| Blood pressure cuffs | $679.40 | 8 BP ($69.95*8) @ $559.60 and 4 CI cuffs ($29.95*4) @ $119.80 |
| Bosch kits | $517.32 |  |
| Dollys | $359.97 | 3 dollys (3*$119.99) |
| In check flowmeters plus mouth pieces | $1,780.00 |  |
| Bluetooth speakers for rehab | $287.30 |  |
| **Facility Rental & Transportation** |  |  |
| Facility rental - Fieldhouse | $8,460.69 | Cost of space rented for COPD rehab program |
| Transportation provided to patients | $1,700.00 | Round trip taxi vouchers provided to patients in need of transportation |
| Total annual cost | $1,498,870.20 |  |
| Patients served | 1,009 |  |
| **Annual cost per patient** | $1,485.50 |  |

Notes:

^a^Payment Schedule for Insured Services Provided by a Physician. Available at https://www.ehealthsask.ca/services/resources/Resources/physician-payment-schedule-oct-17.pdf

^b^All salaries are marked up with a 22.5% for benefits and other costs to the employer. Collective Agreement available at https://www.saho.ca/__media_downloads/HSAS-SAHO-Collective-Bargaining-Agreement-April-2018-to-March-2024-2021-05-25.pdf

^c^Narayen IC, te Pas AB, Blom NA, van den Akker-van ME. Cost-effectiveness analysis of pulse oximetry screening for critical congenital heart defects following homebirth and early discharge. Eur. J. Pediatr. 2019;178:97–103. doi: 10.1007/s00431-018-3268-x.

^d^McAllister, S., Thorn, L., Boladuadua, S. *et al.* Cost analysis and critical success factors of the use of oxygen concentrators versus cylinders in sub-divisional hospitals in Fiji. *BMC Health Serv Res* 21**,**636 (2021). https://doi.org/10.1186/s12913-021-06687-8
